# Impulsivity and Its Relationship With Lisdexamfetamine Dimesylate Treatment in Binge Eating Disorder

**DOI:** 10.3389/fpsyg.2021.716010

**Published:** 2021-08-31

**Authors:** Kristi R. Griffiths, Leonor Aparício, Taylor A. Braund, Jenny Yang, Grace Harvie, Anthony Harris, Phillipa J. Hay, Stephen Touyz, Michael R. Kohn

**Affiliations:** ^1^Brain Dynamics Centre, Westmead Institute for Medical Research, University of Sydney, Westmead, NSW, Australia; ^2^Faculty of Medicine, University of Porto, Porto, Portugal; ^3^BlackDog Institute, Sydney, NSW, Australia; ^4^Faculty of Medicine, University of New South Wales, Sydney, NSW, Australia; ^5^Discipline of Psychiatry, Faculty of Medicine and Health, The University of Sydney, Sydney, NSW, Australia; ^6^School of Medicine, Translational Health Research Institute, Western Sydney University, Sydney, NSW, Australia; ^7^Clinical Psychology Unit, School of Psychology, University of Sydney, Sydney, NSW, Australia; ^8^InsideOut Institute, Charles Perkins Centre, University of Sydney, Sydney, NSW, Australia; ^9^Centre for Research into Adolescents’ Health (CRASH), University of Sydney, Sydney, NSW, Australia; ^10^Adolescent and Young Adult Medicine, Westmead Hospital, Sydney, NSW, Australia

**Keywords:** clinical trial, drug therapy, lisdexamfetamine dimesylate, impulsivity, binge eating disorder

## Abstract

High trait impulsivity is thought to contribute to the sense of loss of control over eating and impulses to binge eat experienced by those with binge eating disorder (BED). Lisdexamfetamine dimesylate (LDX), a drug approved for treatment of moderate to severe BED, has been shown to decrease impulsive features of BED. However, the relationship between LDX-related reductions of binge eating (BE) episodes and impulsivity has not yet been explored. Forty-one adults aged 18–40years with moderate to severe BED completed questionnaires and tasks assessing impulsivity at baseline and after 8weeks of 50–70mg of LDX. Twenty age-matched healthy controls were also assessed at two timepoints for normative comparison. Data were analysed using linear mixed models. BED participants exhibited increased self-reported motor, non-planning, cognitive and food-related impulsivity relative to controls but no differences in objective task-based measures of impulsivity. Food-related and non-planning impulsivity was significantly reduced by LDX, but not to normative levels. Individuals with higher baseline levels of motor and non-planning impulsivity, and loss of control over eating scores experienced the greatest reduction in BE frequency after 8weeks of LDX. Further, there were significant associations between the degree to which subjective loss of control over eating, non-planning impulsivity and BE frequency reduced after 8weeks of LDX. These data suggest that specific subjective measures of impulsivity may be able to predict who will have the greatest benefit from LDX treatment and that reductions in BE frequency may be moderated by concurrent reductions in non-planning impulsivity.

## Introduction

Binge eating disorder (BED) is one of the most prevalent eating disorders worldwide with a lifetime prevalence estimate of 1.9% (Kessler et al., 2013). It is characterised by recurrent episodes of excessive food consumption together with a perceived lack of behavioural control. According to the *Diagnostic and Statistical Manual of Mental Disorders, 5th edition* (DSM-5; American Psychiatric Association, 2013), these binge eating (BE) episodes occur at least once a week for 3months. Furthermore, compensatory purging behaviours to reduce caloric intake as seen in bulimia nervosa are not engaged in regularly in BED. It is generally accepted that the sense of loss of control while eating is the most important and consistent feature of a binge eating episode and leads to marked distress among individuals with BED (Brownstone et al., 2013; Li et al., 2019).

Impulsivity may underlie the loss of control experienced during BE episodes in BED (Dawe and Loxton, 2004; Kessler et al., 2016). Patients suffering from BED show higher general trait impulsivity compared to healthy normal-weight individuals, but potentially also compared to body mass index (BMI)-matched individuals (Giel et al., 2017). Indeed, this trait has been described as a possible hallmark of binge eating behaviour, which is present even in the absence of weight or full-blown eating disorders (Oliva et al., 2019). As a multidimensional and complex construct, impulsivity has distinct neuronal and behavioural components that are differentially disturbed in BED (Dawe and Loxton, 2004; Giel et al., 2017). According to Dawe and Loxton (2004), the main components of impulsivity consist of reward sensitivity and rash-spontaneous impulsiveness. Reward sensitivity refers to the enhanced reward value and selective attention (i.e., attention bias) attributed to food-related cues that prompt the individual to seek appetitive stimuli (Dawe and Loxton, 2004; Hou et al., 2011; Schag et al., 2013). Combined with a reduced delay and probabilistic tolerance (i.e., increased preference for smaller immediate rewards delivered with higher probability over larger delayed rewards delivered with smaller or variable probabilities (Manwaring et al., 2011; Voon et al., 2015), this frequently leads to disadvantageous and impatient decision-making in BED patients. Conversely, rash-spontaneous impulsivity reflects the poor cognitive and motor inhibitory control observed leading up to and during binge episodes (Dawe and Loxton, 2004), which constitutes the diagnostic criteria for BED. More recently, a third domain of impulsivity characterised as the ‘impulsive personality trait’ relates to the persisting underlying tendency to behave impulsively (MacKillop et al., 2016). This differs from ‘state impulsivity’, which can be modulated by external influences (Yeo et al., 2020).

While heightened impulsivity in BED is typically thought to be food-specific, there is evidence of increased impulsive tendencies independent of food cues in people with BED (Schag et al., 2013; Oliva et al., 2020). In an experimental study involving a dice game, Svaldi et al. (2010) concluded that women with BED made more choices that involved larger monetary gains with lower winning probabilities, which reflects higher non-food-specific probabilistic discounting. They further mentioned that women with BED changed their game strategy significantly less often than healthy controls in response to negative feedback after a risky choice (Svaldi et al., 2010), which is consistent with the persistent tendency to make impulsive choices observed in BED. Similarly, individuals with BED chose more often to receive immediate shorter massage time over the same delayed longer reward, also reflecting higher non-food-specific delayed reward discounting (Manwaring et al., 2011). In a motor inhibition task, Mobbs et al. (2011) compared response inhibition towards food- and body-related targets. They found that individuals with BED and a high BMI have a general inhibition problem and difficulty focusing their attention when compared with individuals of normal-weight and without BED, a cognitive deficit that was independent of stimuli type.

There is also a relatively high rate of comorbidity with other impulse control disorders, such as substance use disorders and attention deficit hyperactivity disorder (ADHD; Dawe and Loxton, 2004; Nazar et al., 2016), suggesting common neurobiological underpinnings. Indeed, similar executive function deficits are described in all three aforementioned disturbances: the increased activation of mesolimbic dopaminergic pathway and prefrontal cortex circuits underlies enhanced reward sensitivity and rash spontaneous behaviour (Dawe and Loxton, 2004; Reinblatt, 2015).

Lisdexamfetamine dimesylate (LDX; Vyvanse^®^) is a prodrug of D-amphetamine that is proposed to improve impulse control *via* modulation of corticostriatal circuits, which are broadly involved in reward sensitivity and inhibitory control. LDX was the first approved drug for the treatment of moderate to severe BED in adults and has been shown to not only reduce the intake of highly palatable food in BED models (Presby et al., 2020) but also decrease global binge eating severity and trait impulsive features of BED (McElroy et al., 2016). This is promising, as it suggests that LDX may aid in reducing additional impulse control issues beyond binge eating.

To date, McElroy et al.’s (2016) study is the only study to examine the effects of LDX on impulsivity in BED. While their study provided significant advances in the field, important outstanding questions remain. Firstly, there has been no direct examination of the relationship between LDX-related changes in BE frequency and impulsivity. Second, there has been no comparison with healthy controls to determine whether LDX not only reduces impulsive features, but also normalises them. Finally, McElroy et al. (2016) used self-report measures of trait impulsivity and food-specific impulsivity/compulsivity but no objective task-based measures of impulsivity. Given the recognition of impulsivity as a complex and multifaceted construct, it is important to use various tools to examine the different aspects.

In the present study, we analyse the effects of LDX on different sub-domains of impulsivity in individuals with moderate to severe BED enrolling an open-label phase 4 clinical trial, comparing with healthy controls (HC; Griffiths et al., 2019). Impulsivity was assessed with both subjective and objective measures (Barratt Impulsiveness Scale-11; The Brief Loss of Control over Eating Scale (B-LCOES); and Cued Go No-Go (cGNG) task, Monetary Incentive Delay Task, respectively) that focused on food-specific or non-food-specific aspects. We hypothesised that individuals with BED would have higher levels of impulsivity relative to HC and that LDX would ‘normalise’ impulsivity levels. Given impulsivity is reflective of underlying neurobiology, and LDX treatment targets neurobiology associated with impulsivity; then, greater impulsivity may reflect neurobiological functioning that is more responsive to the benefits of LDX treatment. Therefore, in addition to expecting LDX to reduce binge eating frequency, we hypothesised that impulsivity would moderate the degree of change in binge eating frequency, whereby greater impulsivity would be associated with greater binge eating frequency reductions.

## Materials and Methods

### Participants

Forty-one individuals aged 18–40years, with moderate to severe BED, were recruited *via* referral from participating clinicians or self-referral through online advertisements. All BED participants met the DSM-5 criteria for moderate to severe disease, confirmed by Module I of the *Structural Clinical interview for DSM-5 Research Version* (First et al., 2014). This requires a BE frequency of at least 3days per week in the month prior to the baseline assessment and a minimum score of 4 on the *Clinical Global Impression-Severity* (CGI-S) scale (Busner and Targum, 2007). Inclusion criteria included a BMI between 20 and 45kg/m^2^ and medical approval for LDX commencement.

Twenty age and gender-matched healthy controls (HC) were recruited from the community. They were screened for psychiatric disorders using the *MINI International Neuropsychiatric Interview Version 7.0.2 for DSM-5* (Sheehan et al., 1998; MINI) and excluded if they had any current or past eating disorders. Participants from both groups were excluded if they had certain comorbid psychiatric disorders, such as anorexia nervosa, bulimia nervosa, psychosis, mania and substance dependence; a neurological condition or history of physical brain injury that might interfere with the assessments to be made; and psychostimulant use in the past 6months. Recruitment and testing of all participants occurred from May 2018 to January 2021.

The study was approved by the Human Research Ethics Committee of the Western Sydney Local Health District, and all participants provided written informed consent. The trial was registered at the Australian and New Zealand Clinical Trials Registry (anzctr.org.au) #ACTRN12618000623291.

### Procedure

A description of full trial protocol has been previously reported (Griffiths et al., 2019). Each participant attended a baseline session to complete (1) a clinical interview and health check (2) self-report questionnaires relating to general and food-specific impulsivity, and (3) a series of cognitive tasks. BED participants were provided with a self-monitoring diary and instructed to start LDX 30mg/day. After 2weeks of treatment, the study clinician evaluated them to determine whether it was safe to titrate the dose to 50mg/day. At week 4 of treatment, they were assessed by a study clinician to determine whether the dose should remain at 50mg/day or increase to 70mg/day. At week 8 of LDX treatment, research assessments were repeated, while BED participants were on LDX. HC completed the cognitive tasks at week 8, in order to control for practise effects.

Cognitive tasks were programmed using Inquisit 5 Lab (2018; millisecond.com), and self-report questionnaire data were recorded on RedCap.

### Assessments

#### Binge Eating Frequency

BE frequency was obtained from daily self-monitoring binge eating diaries and confirmed at the baseline and week 8 clinical interviews.

#### Monetary Incentive Delay Task

Reward sensitivity was objectively assessed with the *Monetary Incentive Delay Task* (Knutson et al., 2000). This task consists of multiple trials that require participants to press a button as quickly as possible during the presentation of a visual target, under different monetary reward conditions (potential earning, potential punishment, or no monetary outcome). Incentive task difficulty was calibrated to participants’ mean reaction time (collected before the beginning of the task), so that each participant succeeded on approximately 60% of the incentive trials. Performance feedback appeared immediately after the response and reaction time and accuracy of response (expressed as the percentage of correct responses) were recorded on all trials. Two measures extracted for analysis were as follows: (1) reaction time difference between reward incentive trials and control non-incentive trials, and (2) proportion of accurate reward trials.

#### Cued Go No-Go Task

Rash-spontaneous behaviour was objectively examined with the *Cued Go No-Go task* (Fillmore, 2003), during which participants were asked to quickly respond by pressing a button to go targets and inhibit responding to no-go targets. The task induces response prepotency by presenting a preliminary go or no-go cue before the actual go or no-go target is displayed. The cue-target relationship is manipulated so that in 20% of trials the cue incorrectly signals the target (invalid cue). Percent commission errors (i.e., failure to inhibit response) following a go cue were used to assess the subject’s inhibitory control over a prepotent response.

#### Barratt Impulsiveness Scale-11 (BIS-11)

The *Barratt Impulsiveness Scale* (Patton et al., 1995) is the most widely administered instrument for the assessment of impulsiveness in both research and clinical settings (Stanford et al., 2009). It is a self-report questionnaire that measures both personality and behavioural aspects of impulsivity based on three sub-traits: motor (acting without thinking and inability to concentrate), cognitive (making quick cognitive decisions) and non-planning impulsiveness (lack of forethought; Patton et al., 1995). Each item is answered on a 4-point scale, and then, the sum of the 30 items yields a total impulsivity score that ranges from 30 to 120 (Stanford et al., 2009).

#### The Brief Loss of Control Over Eating Scale

The *Brief Loss of Control over Eating Scale* (Latner et al., 2014) is a 7-item self-reported scale that assesses behavioural, cognitive/dissociative and positive/euphoric aspects related to the loss of control over eating. Each item is rated on a 1–5-point scale with higher scores indicating greater severity of this condition. This measure’s reliability and construct validity are supported by its strong content validity, internal consistency (*α*=0.93), high test–retest reliability (*r*=0.82), and convergent and discriminant validity, when compared to the full 24-item scale (Latner et al., 2014).

### Statistical Analysis

Statistical analyses were designed to address four primary study questions (1) Do individuals with BED have higher levels of impulsivity relative to HC? (2) Does LDX normalise any aberrant measures of impulsivity for individuals with BED? (3) Are LDX-related changes in BE frequency associated with concurrent changes in impulsivity measures? and (4) Do baseline impulsivity levels impact the degree to which LDX reduces BE frequency.

Independent-samples t-tests and ANCOVAs were used first to test whether BED and HC groups differed on baseline demographic and impulsivity measures. Welch two-sample t-tests were conducted in instances with unequal variance between groups.

To evaluate the effects of LDX on outcome measures in the BED group, a linear mixed model was performed separately for each measure, with the impulsivity measure or BE frequency as a dependent variable, individual as a random effect, and timepoint (week 0 and week 8) as a fixed effect. As per previous literature (McElroy et al., 2015), BE frequency was log-transformed to reduce skewness (number of binge eating days per week +1). To determine whether LDX normalised self-report impulsivity measures, independent-samples t-tests were conducted between BED at week 8 and HC at week 0. This was due to self-report questionnaires not being collected for healthy controls at week 8. For cognitive measures, mixed-effect group (BED and HC) by timepoint (week 0 and week 8) interactions were tested to identify if normalisation occurred, while accounting for practise effects.

To assess associations between concurrent changes in impulsivity measures and BE frequency, we included an interaction term to the timepoint model (change in impulsivity × timepoint), with BE frequency as the dependent variable. This model was tested both with and without baseline levels of the impulsivity measures included. Simple effects analyses were used to follow up significant interactions.

To assess whether baseline levels of impulsivity were associated with change in BE frequency, we tested baseline impulsivity × timepoint interactions for each measure, with BE frequency as the dependent variable.

To further explore the relationships between baseline measures of BE frequency and impulsivity, post-hoc correlation analyses were conducted in the BED group. Benjamini-Hochberg corrections were applied to control the false discovery rate.

All analyses were conducted with and without the covariates age, years of education and BMI (covariate-adjusted models reported in supplementary materials). As groups were not matched on BMI, supplementary analyses were also conducted in a BMI-matched sub-sample to determine whether results held. Analyses were performed using R 3.5.1 (Team, 2020). The full sample with baseline data was used to answer question 1 which related to group differences, while questions relating to treatment effects (2,3,4) used only treatment completers. Mixed linear models were tested using the ‘lme4’ package in R (Bates et al., 2015). *p* values for mixed linear models were calculated using the ‘lmerTest’ package in R (Kuznetsova et al., 2017). Cohen’s d effect sizes were calculated using the ‘effectsize’ package in R (Ben-Shachar et al., 2020). Post-hoc effect sizes were calculated using the eff_size function within the ‘emmeans’ package in R (Lenth et al., 2019).

## Results

### Study Population

Forty-one individuals with moderate to severe BED and 20 healthy controls (HC) were assessed at baseline. Thirty-three of the BED participants and 14 HC were assessed at the 8-week follow-up. Baseline demographic and clinical characteristics of recruited individuals in both arms are provided in Table 1. At week 8, 20 BED participants were taking 50mg of LDX, while 13 were taking 70mg. In the BED group, log BE frequency reduced from week 0 (*M*=0.70, *SD* 0.10; mean BE frequency 4.27/week) to week 8 (*M*=0.31, *SD* 0.20; mean BE frequency 1.33/week), [*t*(32)=−9.83, *p*<0.001, *d*=1.71]. This effect remained significant after controlling for baseline log BE frequency, [*t*(69)=−11.98, *p*<0.001].

**Table 1:** Demographics and clinical characteristics.

	BED group (*n*=41)		HC group (*n*=20)	
Age, years M (*SD*)	26.6	5.5	27.5	5.7
**Sex, *n* (%)**				
Female	40	(97.6)	19	(95)
**Race or ethnicity, *n* (%)**				
Caucasian	22	(53.7)	8	(40)
Aboriginal and/or Torres Strait Islander	2	(4.9)	0	(0)
Asian	7	(17.1)	8	(40)
Hispanic	0	(0)	1	(5)
Other or multiple	10	(24.4)	3	(15)
**BMI category, *n* (%)**				
Underweight/normal (<25.0kg/m^2^)	13	(31.7)	12	(60)
Overweight (≥25.0–<30.0kg/m^2^)	16	(39)	7	(35)
Obesity class I (≥30.0–<35.0kg/m^2^)	8	(7.3)	0	(0)
Obesity class II (≥35.0–<40.0kg/m^2^)	3	(7.3)	1	(5)
Obesity class III (≥40.0kg/m^2^)	1	(2.4)	0	(0)
**Current psychiatric comorbidities, *n* (%)**				
Major depressive disorder	3	(7.3)	0	(5)
Generalised anxiety disorder	2	(4.9)	0	(0)
Social anxiety disorder	3	(7.3)	0	(0)
Obsessive–compulsive disorder	1	(2.4)	0	(0)
Alcohol use disorder	6	(14.6)	0	(0)
Substance use disorder	2	(4.9)	0	(0)
Adult ADHD	5	(12.2)	0	(0)

*M, mean; SD, standard deviation; n, number; BMI, body mass index; and ADHD, attention deficit hyperactivity disorder*.

### Individuals With BED Had Higher Levels of Self-Reported Impulsivity Relative to HC, but Not Task-Based Measures of Impulsivity

**Figure 1:**
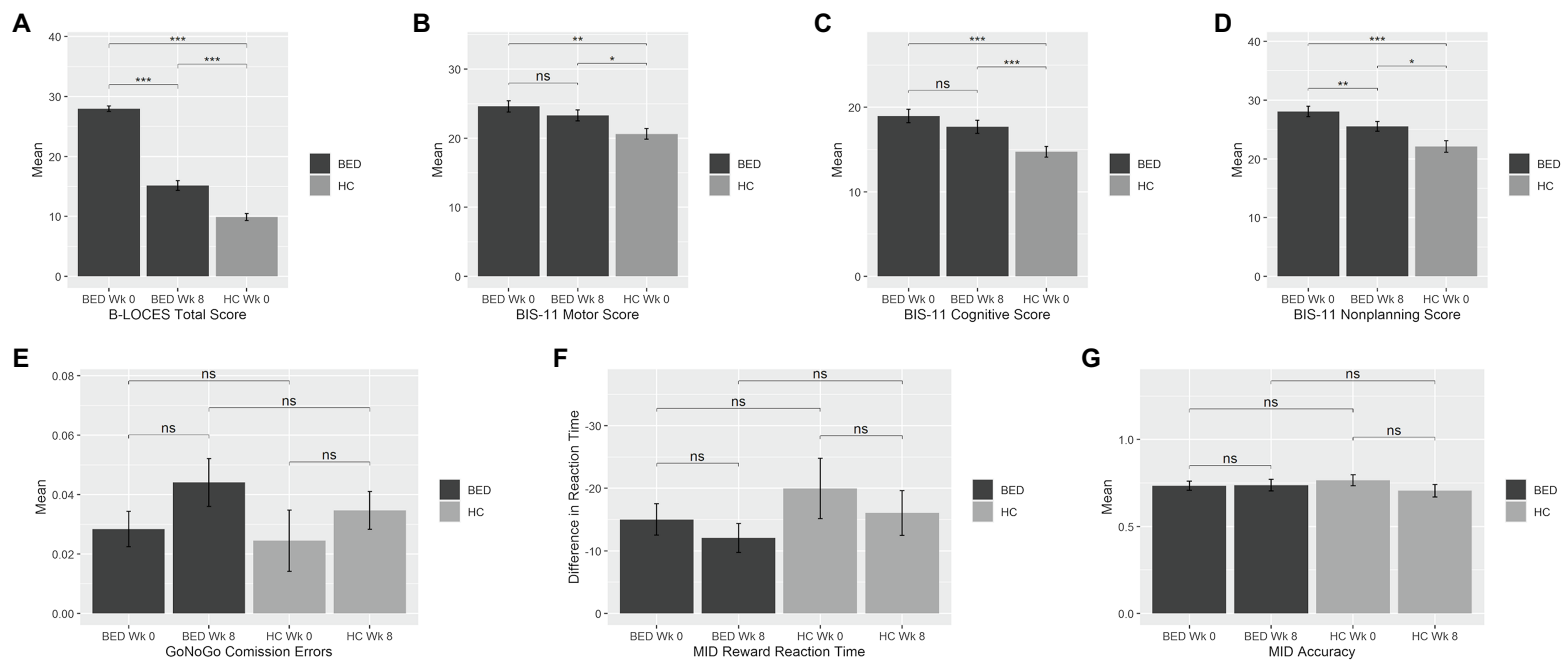
Mean impulsivity measures at week 0 and week 8 for the binge eating disorder (BED) and healthy control (HC) groups. Bars represent means, and error bars represent standard error from the mean. BED, Binge Eating Disorder; HC, healthy control; Wk, week; BIS, Barratt Impulsiveness Scale; MID, Monetary Incentive Delay. BED, Binge Eating Disorder; HC, healthy control; Wk, week; BIS, Barratt Impulsiveness Scale; MID, Monetary Incentive Delay. *p=0.05; **p=0.001; ***p<0.001.

At baseline, BED participants had higher scores than HC on the B-LOCES [BED, *M* 27.95, *SD* 2.98; HC, *M* 9.89, *SD* 2.62; *t*(58)=22.62, *p*<0.001, *d*=6.43], BIS-11 motor [BED, *M* 24.61, *SD* 5.21; HC, *M* 20.63, *SD* 3.37; *t*(58)=3.04, *p*=0.004, *d*=0.91], BIS-11 cognitive [BED, *M* 18.98, *SD* 5.05; HC, *M* 14.74, *SD* 2.85; *t*(55.59)=4.14, *p*<0.001, *d*=1.03] and BIS-11 non-planning scales [BED, *M* 28.07, *SD* 5.64; HC, *M* 22.11, *SD* 4.32; *t*(58)=4.09, *p*<0.001, *d*=1.19; see Figure 1].

**Figure 2:**
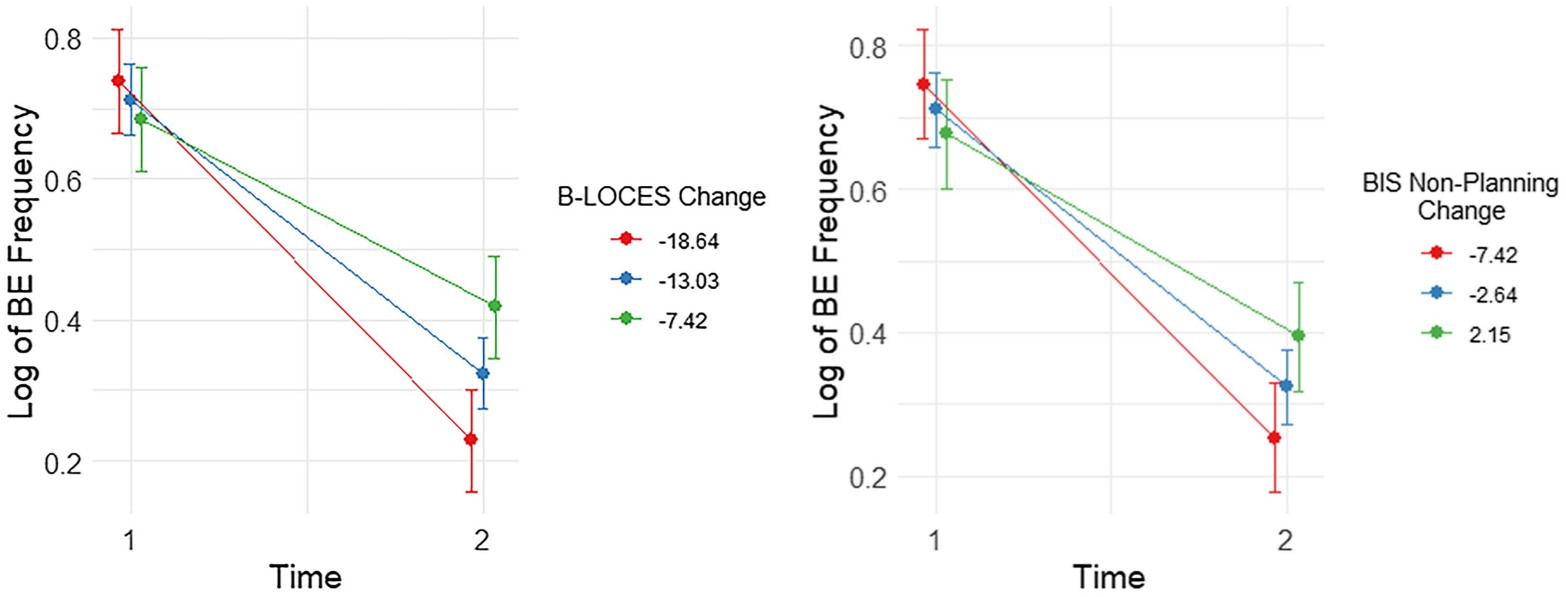
Plots showing significant interactions between change in Log Binge Eating (BE) Frequency and Brief Loss of Control over Eating Scores (B-LOCES) and BIS non-planning from time 1 to 2. Following the convention suggested by Aiken and West (1991), we used the mean value of the moderators (i.e. change in BIS non-planning and B-LOCES scores) as well as one standard deviation below and above the mean value to plot the moderating effect of these measures on BE frequency between baseline and week 8.

Groups did not significantly differ in cGNG percent commission errors during go-cue/nogo-target trials [BED, *M* 2.84, *SD* 3.82; HC, *M* 2.45, *SD* 4.61; *t*(56)=0.35, *p*=0.731], in MIDT reward reaction time[(BED, *M* -15.02, *SD* 15.97; HC, *M* -19.98, *SD* 21.60; *t*(55)=0.99, *p*=0.328] or reward trial accuracy [BED, *M* 0.73, *SD* 0.17; HC, *M* 0.77, *SD* 0.14; *t*(55)=−0.72, *p*=0.475].

### Eight Weeks of LDX Reduced but Did Not Normalise Aberrant Measures of Self-Reported Impulsivity For Individuals With BED

After 8weeks of LDX, the BED group reported reductions in B-LOCES [week 0, *M* 27.95, *SD* 2.98; week 8, *M* 15.15, *SD* 5.30; *t*(32)=−13.24, *p*<0.001, *d*=2.3] and BIS-11 non-planning [week 0, *M* 28.07, *SD* 5.64; week 8, *M* 25.55, *SD* 5.33; *t*(32.64)=−3.14, *p*=0.004, *d*=0.55] relative to baseline; however, both measures remained elevated relative to HC [B-LOCES, *t*(49.18)=4.77, *p*<0.001, *d*=1.26; BIS-11 non-planning, *t*(50)=2.39, *p*=0.021, *d*=0.71].

There were no significant LDX-related changes in BIS-11 motor [week 0, *M* 24.61, *SD* 5.21; Week 8, *M* 23.30, *SD* 4.97; *t*(32)=−1.87, *p*=0.071] or BIS-11 cognitive [week 0, *M* 18.98, *SD* 5.05; week 8, *M* 17.70, *SD* 5.03; *t*(32)=−1.51, *p*=0.139], and these measures remained elevated relative to HC [BIS-11 motor, *t*(50)=2.08, *p*=0.043, *d*=0.63; BIS-11 cognitive, *t*(50)=2.71, *p*=0.009, *d*=0.73].

Cued go no-go task percent commission errors did not change significantly [week 0, *M* 2.84, *SD* 3.82; week 8, *M* 4.40, *SD* 5.16; *t*(31)=−1.69, *p*=0.101]. Similarly, the BED group did not experience significant change in MIDT reward reaction time from week 0 to week 8 [week 0, *M* -15.02, *SD* 15.97; week 8, *M* -12.06, *SD* 14.88; *t*(35.72)=0.78, *p*=0.440] or reward trial accuracy [week 0, *M* 0.73, *SD* 0.17; week 8, *M* 0.74, *SD* 0.21; *t*(32.65)=−0.19, *p*=0.852]. There were no significant timepoint x group interactions in MIDT reward reaction time [*t*(53.55)=0.32, *p*=0.752] or reward trial accuracy [*t*(48.88)=−1.27, *p*=0.212], or cGNG percent commission errors [*t*(55.60)=−0.32, *p*=0.753].

The inclusion of age, education and BMI as covariates did not alter any of these findings.

### LDX-Related Reductions in BE Frequency Were Associated With Concurrent Changes in BIS Non-planning and B-LOCES Scores

There were significant interactions between change in log BE frequency and change in BIS non-planning, [*t*(31.00)=2.96, *p*=0.006] and change in B-LOCES, [*t*(31.00)=3.59, *p*=0.001]. These effects remained significant after controlling for baseline BIS non-planning, [*t*(31.00)=2.96, *p*=0.006] and baseline B-LOCES, [*t*(31.00)=3.59, *p*=0.001], respectively.

Simple effects analysis showed that reductions in log BE frequency from week 0 to week 8 were most pronounced for those with the largest reductions in BIS-11 non-planning (i.e., around −7.42 reduction, *b*=−0.50, *t*(31)=−9.78, *p*<0.001, *es*=3.43), though still quite pronounced with smaller reductions (i.e., around −2.64 reduction, *b*=−0.39, *t*(31)=−10.97, *p*<0.001, *es*=2.70) and small increases (i.e., around 2.15 increase, *b*=−0.28, *t*(31)=−5.57, *p*<0.001, *es*=1.96; Figure 2).

**Figure 3:**
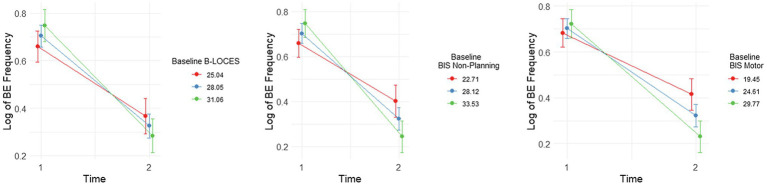
Plots showing significant interactions between change in Log Binge Eating (BE) Frequency from time 1 to 2 and baseline (i.e., time 1) levels of Brief Loss of Control over Eating Scores (B-LOCES), BIS non-planning and BIS motor scores. Following the convention suggested by Aiken and West (1991), we used the mean value of the moderators (i.e., baseline B-LOCES, BIS non-planning and BIS motor scores) as well as one standard deviation below and above the mean value to plot the moderating effect of these measures on BE frequency between baseline and week 8.

Simple effects analysis also showed that the reduction in log BE frequency from week 0 to week 8 was most pronounced for those with the largest reductions in B-LOCES [i.e. around −18.64, *b*=−0.51, *t*(31)=−10.67, *p*<0.001, *es*=3.72], but also for those with average reductions in B-LOCES [i.e. around −13.03, *b*=−0.39, *t*(31)=−11.53, *p*<0.001, *es*=2.84], and small reductions in B-LOCES [i.e. -7.42, *b*=0.27, *t*(31)=5.57, *p*<0.001, *es*=1.95; Figure 2.]

There were no significant interactions between change in log BE frequency and change in BIS motor [*t*(31.00)=1.52, *p*=0.139], change in BIS cognitive [*t*(31.00)=1.97, *p*=0.058], change in cGNG commission errors [*t*(30.00)=0.78, *p*=0.442], change in MIDT reward reaction time [*t*(56.00)=0.11, *p*=0.915] or MIDT reward trial accuracy [*t*(56)=−0.38, *p*=0.703]. These results did not change when controlling for baseline BIS motor, [*t*(61)=1.53, *p*=0.139], baseline BIS cognitive score, [*t*(31.00)=1.97, *p*=0.058], baseline cGNG commission errors, [*t*(30.00)=0.78, *p*=0.442], baseline MIDT reward reaction time [*t*(28.00)=0.11, *p*=0.915] or baseline MIDT reward accuracy [*t*(28.00)=−0.38, *p*=0.710], respectively.

### Baseline BIS Motor, BIS Non-planning and B-LOCES Scores Moderated the Degree to Which LDX Reduced BE Frequency

There was a significant interaction between change in log BE frequency and baseline BIS motor score, *t*(38.56)=−3.48, *p*=0.001, BIS non-planning score, *t*(41.05)=−3.84, *p*<0.001, and B-LOCES, *t*(38.07)=−2.48, *p*=0.018.

Simple effects analysis also showed that reduction in log BE frequency from week 0 to week 8 was most pronounced for those with the highest BIS non-planning scores at baseline [i.e. around 33.53, *b*=−0.50, *t*(37.3)=−11.20, *p*<0.001, *es=* 3.80], followed by those with average BIS non-planning scores [i.e. around 28.12, *b*=−0.38, *t*(36.1)=−12.10, *p*<0.001, *es=* 2.87] and those with the lowest BIS non-planning scores [i.e. 22.70, *b*=−0.26, *t*(38.0)=−5.68, *p*<0.001, *es=* 1.94; Figure 3].

Similarly, reduction in log BE frequency from week 0 to week 8 was most pronounced for those with the highest baseline B-LOCES scores [i.e. around 31.06, *b*=−0.46, *t*(34.6.0)=−9.59, *p*<0.001, *es=* 3.18], followed by those with average B-LOCES scores [i.e. around 28.05, *b*=−0.39, *t*(36.6)=−11.00, *p*<0.001, *es=* 2.59] and those with the lowest B-LOCES scores [i.e. 25.04, *b*=−0.29, *t*(37.5)=−5.98, *p*<0.001, *es=* 2.00; Figure 3.]

Finally, reduction in log BE frequency from week 0 to week 8 was also most pronounced for those with the highest BIS motor scores [i.e. around 29.77, *b*=−0.49, *t*(36.6)=−10.75, *p*<0.001, *es=* 3.60], followed by those with average BIS motor scores [i.e. around 24.61, *b*=−0.38, *t*(36.5)=−11.78, *p*<0.001, *es=* 2.78] and those with the lowest BIS motor scores [i.e. 19.45, *b*=−0.27, *t*(36.2)=−5.83, *p*<0.001, *es=* 1.95; Figure 3.]

Change in BE frequency did not interact significantly with baseline BIS cognitive scores, *t*(40.53)=−1.42, *p*=0.164, cGNG commission errors, *t*(38.05)=−1.26, *p*=0.214, MIDT reward reaction time, *t*(65)=−0.49, *p*=0.638, or MIDT reward trial accuracy, *t*(65)=−0.24, *p*=0.816.

### Baseline BE Frequency Was Positively Correlated With B-LOCES and BIS Non-planning in the BED Group

In the BED group, log BE frequency was positively correlated with B-LOCES (*r*=0.44, *p*=0.004) and BIS-11 non-planning (*r*=0.46, *p*=0.002). B-LOCES was positively correlated with BIS-non-planning (*r*=0.421, *p*=0.006). No other correlations survived correction for multiple comparisons (*q*=0.0062). No correlations were observed in the baseline impulsivity measures for the HC group.

## Discussion

In the current study, we investigated impulsivity in moderate to severe BED and its relationship with LDX efficacy. We found that individuals with BED reported increased food-specific and general impulsivity on self-report measures relative to controls, but no differences in task-based measures. Eight weeks of LDX treatment reduced food-specific and a ‘non-planning’ scale of impulsivity, but did not normalise these measures. However, the degree of reduction in these two measures was associated with the level of concurrent reductions in BE frequency after LDX treatment. Finally, individuals with higher baseline levels of food-related, non-planning and motor impulsivity experienced the greatest reductions in BE frequency after 8weeks of LDX.

Consistent with previous research, the BED group had elevated levels of self-reported impulsivity relative to HC in both food-specific and general measures of impulsivity (Meule, 2013; Bodell et al., 2018). This increase in more general impulsivity is particularly interesting as it suggests that individuals with BED may experience challenges with behaviours beyond eating. Indeed, there is a body of research into the increased co-occurrence of binge eating with ADHD (Cortese et al., 2007; Derefinko et al., 2008), problem gambling (Jiménez-Murcia et al., 2013; Farstad et al., 2015) and substance abuse (Bahji et al., 2019), which may all stem from this impulsive behavioural phenotype. This is further supported by descriptions of shared pathophysiological mechanisms underlying these psychiatric entities (Dawe and Loxton, 2004; Schreiber et al., 2013; Reinblatt, 2015), which opens up opportunities to study and manage patients with co-morbid and interrelated impulsivity disturbances with a single treatment.

Despite some previous research reporting that people with BED have an increased tendency to act rashly and spontaneously in inhibitory control tasks (Schag et al., 2013; Wu et al., 2013; Giel et al., 2017), we did not find differences in measures assessed from the Go-NoGo and monetary incentive delay tasks. One possible reason for this discrepancy is that most previous studies focused on people with BED and a BMI over 30 (Monica et al., 2010; Mobbs et al., 2011; Loeber et al., 2012; Wu et al., 2013; Hege et al., 2015), whereas 70% of our BED cohort were in the normal or overweight BMI range (BMI <30kg/m^2^). Nonetheless, controlling for BMI statistically did not alter our findings. In addition, a number of studies used food-related versions of the Go-NoGo, which may increase task salience in the BED group, thereby strengthening group differences. However, other studies also found no differences in food-related response inhibition between groups (Svaldi et al., 2015), revealing the inconsistent nature of inhibitory control task results in people with BED. As suggested by Kollei et al. (2018), it is possible that state-based factors, such as hunger or stress, could moderate performance, and should be measured in future studies.

Impulsivity is not a unidimensional concept itself, so different modalities of measurement (e.g., self-report *versus* objective behavioural tasks) may assess different aspects of inhibitory control (Dawe and Loxton, 2004; Giel et al., 2017). The proposed ‘trait *versus* state dichotomy of impulsivity’ (Yeo et al., 2020) suggests that self-report measures, like the BIS-11, capture a more trait-like representation of impulsivity, while behavioural tasks may be more dependent on state-dependent factors, such as stress. Further, neuroimaging research from children with ADHD shows that psychostimulants may be most effective at ameliorating aberrances in reward and inhibitory control circuits (Solanto, 1998), which are more likely to present as changes in trait-like impulsivity than environmentally-induced state factors. In line with previous studies demonstrating low correlations between trait and state measures of impulsivity (Wingrove and Bond, 1997; Aichert et al., 2012), it seems that cGNG and BIS-11, especially the motor subscale, do not measure the same aspects of impulsivity. This difference may thus reflect an active compensatory effort to slow down responses in behavioural tasks after realising that they tend to behave impulsively (Wingrove and Bond, 1997).

After 8weeks of LDX intervention, participants with BED reported a significant reduction not only in BE frequency, but also in B-LOCES total and BIS-11 non-planning scores. This largely supports the findings by McElroy et al. (2016); however, we did not replicate their finding of reductions in the BIS motor subscale. This may be due to McElroy et al. assessing BED patients after 11weeks of LDX treatment relative to 8weeks of treatment in the current study. Despite the significant reduction that was found, both measures remained elevated compared to controls, which suggests a decreasing trend in general and food-specific self-reported impulsivity measures that does not reach normalised levels after 8weeks of LDX treatment. McElroy et al. (2016) did show continued reductions in most measures between weeks 8 and 11 in their LDX efficacy trial; therefore, it is plausible that continued use would have led to significantly reduced BIS motor scores. Overall, these results demonstrate that LDX plays an important role in self-perceived impulsiveness beyond that relating to food. Further research would be beneficial in evaluating the broader impact of LDX in the subset of patients with comorbid issues relating to impulse control.

Two measures stood out in their strong correlation with BE frequency, both at baseline and with regard to treatment-related change: B-LOCES and BIS non-planning. This is somewhat unsurprising for the B-LOCES and is consistent with the aforementioned role of the sense of loss of control while eating in BED characterisation and severity. However, it is less obvious why BIS non-planning is so tightly coupled to BE frequency in a BED sample. The fact that participants with BED scored higher in items like ‘I do things without thinking’ and ‘I am more interested in the present than in the future’ suggests that they have a present orientation that interferes with eating patterns. This is supported by the positive correlation between BIS-11 non-planning and B-LOCES, suggesting that individuals who are less able to plan ahead may end up experiencing greater loss of control during binges. Together with the positively correlated BIS-11 non-planning and BE frequency, it reinforces the idea that BED severity is influenced by different time-spaced components of impulsivity.

Despite the overall high efficacy of LDX in reducing BE frequency, there was some individual variability in the degree to which this change occurred. Our data showed that individuals with higher baseline levels of motor and non-planning impulsivity and B-LOCES scores experienced the greatest reduction in BE frequency during 8weeks of LDX. This has important clinical implications, as it means that two brief and easy to administer questionnaires may be useful for predicting who will benefit the most from LDX treatment. It is known that psychological therapies do not have the same level of efficacy for all people with BED (Hilbert et al., 2019). Individuals with particularly high levels of motor and non-planning impulsivity may represent a subgroup of people with BED who would benefit from LDX as an adjunct to psychological therapy.

Some limitations of this study should be acknowledged. First, healthy control participants were not matched to BED group in terms of BMI. This is potentially important given that previous research has reported increased impulsivity in people with obesity, in the absence of BED (Derefinko et al., 2008). However, the inclusion of BMI as a covariate of no interest in all analyses (supplementary materials) did not alter any of the results, suggesting that group differences were due to psychological rather than weight-based factors. Second, this trial was not designed to be an efficacy study and therefore does not include a placebo arm. As such, we cannot conclusively say that reductions in BE frequency and impulsivity measures are due to LDX treatment alone. It does however replicate the real-world setting where placebo and physician contact effects contribute to the overall clinical response. Further, this study focussed on the relationships between impulsivity and BE frequency measures, which we can assume were equally affected by placebo effects. Third, the BIS-11 and B-LOCES were not collected at week 8 for control participants. Although no major changes in self-perceived impulsivity were expected to have happened during this period for healthy controls, we cannot rule out that effects seen in the BED group were in part due to time-related factors rather than LDX. Given the significant correlations between baseline measures, this seems unlikely. Finally, only one male BED participant was recruited into the study, despite the fact that BED has the highest male-to-female ratio of any eating disorder (Hudson et al., 2007). This can likely be attributed to the increased stigma around BED in men (Thapliyal et al., 2018), and lower health-seeking behaviour rates of men relative to women (Kessler et al., 2013). There are documented differences between males and females in impulsive action and choice (Weafer and de Wit, 2014), which unfortunately limits the generalisability of these results to male BED patients.

To conclude, self-perceived impulsivity features are significantly associated with BED psychopathology and are susceptible to LDX-induced changes. This has broad clinical implications, such as the ability to predict response to LDX in moderate to severe BED, and potential use in concurrently treating a range of comorbid impulse control disorders or addictive behaviours. Finally, future research should be conducted to investigate whether LDX-induced reductions in trait impulsivity may enhance treatment outcomes for additional treatments, such as cognitive behavioural therapy.

## Data Availability Statement

The raw data supporting the conclusions of this article will be made available by the authors, without undue reservation.

## Ethics Statement

The studies involving human participants were reviewed and approved by Western Sydney Local Health District. The patients/participants provided their written informed consent to participate in this study.

## Author Contributions

KG designed the study protocol, collected and analysed the data, and wrote the paper. LA collected and analysed the data and wrote the paper. TB analysed the data and wrote the paper. JY and GH collected the data and wrote the paper. AH assessed the clinical patients and wrote the paper. PH and ST designed the study protocol and wrote the paper. MK designed the study protocol, assessed the clinical patients and wrote the paper. All authors contributed to the article and approved the submitted version.

## Conflict of Interest

KG has been the recipient of honoraria from Shire (Takeda group of companies) for public speaking engagements. PH receives/has received sessional fees and lecture fees from the Australian Medical Council, Therapeutic Guidelines publication, and HETI (New South Wales and the former NSW Institute of Psychiatry) and royalties/honoraria from Hogrefe and Huber, McGraw Hill Education, and Blackwell Scientific Publications, Biomed Central and PlosMedicine, and she has received research grants from the NHMRC and ARC. She is Chair of the National Eating Disorders Collaboration Steering Committee in Australia (2019-) and was Member of the ICD-11 Working Group for Eating Disorders and was Chair Clinical Practice Guidelines Project Working Group (Eating Disorders) of RANZCP (2012â€“2015). She has prepared a report under contract for Takeda (formerly Shire) Pharmaceuticals in regard to binge eating disorder (July 2017) and is a consultant to Takeda Pharmaceuticals. All views in this paper are her own. ST has been the recipient of honoraria from Shire (Takeda group of companies) for public speaking engagements and commissioned reports. He has been the Chair of their Clinical Advisory Group for binge eating disorder. He receives honoraria for books/book chapters from McGraw Hill. Hogrefe and Huber and Taylor and Francis. He is the co-director of the Inside Out Institute at the University of Sydney. ST is a mental health consultant to BUPA. He is a member of the Department of Health Technical Advisory Group for Eating Disorders (Commonwealth Government). MK has been the recipient of honoraria from Shire (Takeda group of companies) for public speaking engagements. LA, TB, JY, GH and AH have no potential conflicts of interest to declare.

## Publisher’s Note

All claims expressed in this article are solely those of the authors and do not necessarily represent those of their affiliated organizations, or those of the publisher, the editors and the reviewers. Any product that may be evaluated in this article, or claim that may be made by its manufacturer, is not guaranteed or endorsed by the publisher.

## Abbreviations

Abbreviations
B-LOCES: Brief Loss of Control Over Eating Scale
BE: binge eating
BED: binge eating disorder
BIS-11: Barratt Impulsiveness Scale-11
cGNG: Cued Go No-Go
LDX: Lisdexamfetamine dimesylate
MIDT: Monetary Incentive Delay Task
